# Building systems’ resilience in the mpox outbreak response in Africa

**DOI:** 10.4102/jphia.v16i1.875

**Published:** 2025-03-31

**Authors:** Ngashi Ngongo, Nicaise Ndembi, Tajudeen Raji, Mosoka Fallah, Wessam Mankoula, Jean-Marie Vianny Yameogo, Fiona Braka, Abdou Salam Gueye, Matshidiso R. Moeti, Jean Kaseya

**Affiliations:** 1 Africa Centres for Disease Control and Prevention (Africa CDC), Addis Ababa, Ethiopia; 2 World Health Organization, African Region (WHO AFRO), Brazzaville, Republic of the Congo

On 05 May 2023, the World Health Organization (WHO) declared the end of mpox as a public health emergency of international concern (PHEIC). Just 15 months later, Africa is battling the mpox outbreak again, following the declaration by both Africa Centres for Disease Control and Prevention (CDC) and WHO as a public health emergency of continental security on 13 August 2024, and a PHEIC on 14 August 2024.^1,2^ At the time of the declarations in mid-August 2024, Africa had recorded 17 541 cases and 517 deaths from 13 affected countries.^3^ Six weeks after the declarations, the number of cases increased rapidly by 82% to reach 32 010 cases and 840 deaths reported from 15 countries. Compared to the same period in 2023, this represents 195% increase in the number of cases and 39% in the number of deaths.

The rapid increase in the number of cases reported is facilitated by weaknesses in surveillance, laboratory testing, community engagement, infection prevention and control (IPC) and isolation and case management. This increased reporting may also be partially linked to better surveillance and awareness. In that regard, the current surveillance is predominantly passive with most cases being identified during normal consultations and low rate of contact follow-up estimated around 3%; the laboratory capacity is low with testing and positivity rates estimated at 49% (Range: 38.6% – 100%) and 40% (Range: 0.3% – 55%), community health workers (CHWs), village health committees and community-based organisations are not actively involved in the response, the IPC assessment in 17 African countries conducted from 2020 to 2022 revealed a score of 56% in primary healthcare (PHC) facilities.^4^ In addition, frequent lapses in personal protective equipment (PPE) have been reported often because of inadequate human resources, knowledge and supplies.^5^ Furthermore, the lack of food provision in the mpox treatment centres for those caring for the sick has led to situations where these persons go out to the communities and public markets searching for food with the risk of spreading the mpox virus.

Over the last decade, Africa has experienced a major outbreak every 3 years, starting with the major Ebola outbreak in West Africa in 2014–2016, the Ebola outbreak in the Democratic Republic of the Congo (DRC) 2018–2019, coronavirus disease 2019 (COVID-19) pandemic in 2020–2022 and mpox outbreak in 2023–2024. The severity and duration of these health emergencies were dependent on the strength of the health systems of affected countries. The same health system weaknesses observed in the current mpox outbreak were reported in previous emergencies, in Ebola and in COVID-19.^6,7,8^ In addition, the most recent readiness assessments conducted by Africa CDC and the WHO both confirmed that African countries do not have adequate capacity to prevent and rapidly control outbreaks.^4^ Unfortunately, the same observations are made every time an outbreak strikes: the surveillance system is weak and passive, contact tracing is not systematically done, laboratory testing capacity is weak, emergency sample transportation systems are not in place, IPC measures are lacking both at community and health facility levels and isolation facilities are not adequate. The focus has always been on the immediate emergency response with little attention on strengthening the health system resilience.

The Ebola outbreak in West Africa was both a wakeup call and a missed opportunity. An estimated $6 billion was spent to fight the Ebola Virus Disease (EVD) in West Africa in 2014–2016^9^ and more than $1 billion in the DRC alone during the 2018–2020 outbreak.^10^ In addition, the COVID-19 pandemic mobilised substantive funding, including $63 billion committed by African countries in their national budgets to fight COVID-19 in 2020–2021.^11^ Today, most of the investments have been wiped away, leaving no trace behind. Among the few visible legacies of the COVID-19 pandemic is the strengthening of laboratory sequencing capacity which showed significant improvements from just two countries with at least one laboratory equipped to perform pathogen sequencing to about 40 countries at the time of the declaration of the end of COVID-19 as a PHEIC.

The Mpox Continental Incident Management and Support Team (IMST) is coordinating the mpox preparedness and response with a plan budgeted at $599 million. The continental plan has prioritised four main legacy initiatives: (1) decentralisation of the digital transformation of the PHC surveillance and information systems, (2) decentralisation of the laboratory diagnostics capacity, (3) scale up of CHWs towards the 2 million target set by the African heads of state and government and (4) strengthening of the IPC in health facilities and in schools.

*Digital transformation of PHC surveillance and information systems*: The timely availability of quality surveillance data has been a major challenge in previous outbreaks, including EVD and COVID-19.^12,13^ The same challenges have been observed during the current mpox outbreak in the affected countries. Several digital technologies have been used in community surveillance of notifiable diseases, One Health, human immunodeficiency viruses (HIV), acquired immunodeficiency syndrome (AIDS) and tuberculosis, with the aim to improve the timeliness of data availability to facilitate the early disease detection and response.^14,15^ Building on these experiences, the Continental IMST has identified digital transformation of the surveillance and information systems as an important legacy of the mpox response that will speed up the control of the current outbreak and strengthen the resilience of health systems of affected countries to prepare them for future public health threats. This will include scaling up digital technologies in the health facilities and at community level in the hands of CHWs, linked to the District Health Information System 2 (DHIS2) tracker. In addition, interconnectivity will be strengthened between DHIS2 tracker, mpox testing laboratories and treatment centres to link the surveillance data with laboratory results and treatment outcomes. This is an important building block in our effort to strengthen pandemic prevention, preparedness and response.

*Strengthening laboratory testing capacity for diagnosis and pathogen sequencing*: In an outbreak, delays to confirm the diagnosis keep the person suspected of having acquired the infection at large to continue living in the same household and community, spreading the disease to their close contacts. Therefore, strengthening laboratory testing capacities at all levels, with particular attention to the decentralised level, is an important area of investment and one that can have lasting positive impacts. In the context of the mpox outbreak response, the continental IMST recommends a five-pronged strategy to: (1) improve the skills of frontline health workers in sample collection, storage and packaging for transportation, (2) set up an emergency integrated sample transportation system from health facilities to the designated laboratories organised in clusters, (3) decentralise the laboratory diagnostic capacity placing GeneXpert machines with cartridges in every health district and qPCR (quantitative polymerase chain reaction) machines with diagnostic kits in provincial laboratories, (4) strengthen pathogen sequencing capacity in national laboratories and (5) reinforce the biosafety and biosecurity at all levels. Once accomplished, affected countries will have sufficient diagnostics and sequencing capacities to stop the current mpox outbreak and prepare for future public health emergencies.

*Scale up of CHWs skilled and tooled in surveillance, risk communication and community engagement (RCCE) and home isolation and care*: For many communities, CHWs constitute the first point of contact with the health system for health education and access to basic health services.^16^ In outbreak preparedness and response, CHWs have played a vital role in active case search through door-to-door home visits to screen, identify and refer individuals meeting the mpox case definition to appropriate healthcare facilities.^17^ In addition, CHWs have been instrumental in the identification and follow up of contacts during the incubation period as well as enhancing the community awareness on the vaccination strategies. Furthermore, they have helped in home isolation and care. These are the same roles they are expected to play in the current mpox response. Therefore, the continental IMST is supporting countries to scale up remunerated CHWs to ensure adequate geographical coverage, with particular attention to the most vulnerable and hard-to-reach regions. In this regard, we recommend a shift towards an integrated modular training of CHWs with tools to give them the needed skills in surveillance, IPC and RCCE. To ensure sustainability, governments must commit to take up the remuneration of CHWs in their national budgets as partners cannot bear recurrent costs for ever. The experience of countries such as Ethiopia has demonstrated that it is possible.^18^ At the beginning, partners could commit to fund the initial years as governments progressively take over and absorb the remuneration of CHWs in national and local budgets as well as through revenues generated by local authorities. The scale up of CHWs trained, supported and remunerated is a key legacy of the mpox outbreak response.

*Permanent IPC facilities in health facilities and schools*: Frontline health workers are often the first victims of major infectious disease outbreaks in Africa for reasons that include close contacts with infected patients, delayed diagnosis and weak adherence to IPC measures.^19,20^ Past outbreaks have shown that unprepared health facilities with weak IPC measures constituted an important source of the spread of infectious diseases during outbreaks.^21,22^ Likewise, there is evidence that schools play a role in the spread of infectious diseases during outbreaks.^23^ In that regard, one common immediate action after the declaration of major outbreaks has been to close schools to protect the children against the risk of acquiring the disease.^24^ The gathering of children in schools during a public health emergency caused by an infectious disease constitutes a major risk for the further spread because of the prolonged physical exposure during intra and extramural school activities. Therefore, schools may become a safe haven for children if the basics of disease surveillance and IPC can be established. Thinking in the long term, unlike in the past where short-term solutions were sought (e.g., plastic buckets for handwashing), the current mpox preparedness and response provides an opportunity for constructing permanent water, sanitation and hygiene facilities, and changing the hygiene practices in schools and in health facilities. In schools, preference should be given to permanent water tanks with cement-based facilities that allow children to wash their hands when they arrive and before they leave. Instilling good hygiene habits in children from an early age contributes to their education and sets an example for the future. In addition, schools must have at least one nurse on duty every day, and all teachers could be trained and transformed into surveillance officers able to screen, identify sick children, and refer them for further investigation and management. Furthermore, basic triage and isolation facilities should become a standard in every health facility from the health centre to the referral hospital, and hospitals must have permanent isolation facilities for contagious diseases that could be activated in case of an outbreak.

There is wide consensus among experts to build the resilience of health systems. However, it has remained an unfulfilled promise. The current mpox outbreak preparedness and response has brought many innovations with the continental IMST that is a unique and first of its kind model of an emergency response coordination in Africa. Learning from the weaknesses of the past, the current mpox outbreak response offers an opportunity to invest in health systems strengthening from the start, during the planning phase to take advantage of the first resources to include a legacy agenda. Each of the four resilience legacy initiatives discussed earlier has some successful experiences and lessons learned on which to build the learning. The excuses of the past won’t hold this time. The accountability rests on the health leadership in affected countries and the continental IMST to manage the current emergency response differently.
